# Effects of nano-selenium on cecum microbial community and metabolomics in chickens challenged with Ochratoxin A

**DOI:** 10.3389/fvets.2023.1228360

**Published:** 2023-09-05

**Authors:** Manxin Fang, Wei Hu, Ben Liu

**Affiliations:** ^1^College of Life Science and Resources and Environment, Yichun University, Yichun, China; ^2^Engineering Technology Research Center of Jiangxi Universities and Colleges for Selenium Agriculture, Yichun University, Yichun, China

**Keywords:** nano-selenium, cecum microbial community, metabolomics, chickens, Ochratoxin A

## Abstract

**Introduction:**

Ochratoxin A (OTA) is a widely distributed mycotoxin. Nano-selenium (Nano-Se) is an emerging form of selenium known for its superior bioavailability, remarkable catalytic efficiency, and robust adsorbing capacity. Despite these characteristics, its impact on the microbial community and metabolomics in the cecum of chickens exposed to OTA has been infrequently investigated. This research examined the microbiota and metabolomic alterations linked to OTA in chickens, with or without Nano-Se present.

**Methods:**

A cohort of 80 healthy chickens at the age of 1 day was randomly distributed into four groups of equal numbers, namely the Se cohort (1 mg/kg Nano-Se), the OTA cohort (50 μg/kg OTA), the OTA-Se cohort (50 μg/kg OTA + 1 mg/kg Nano-Se), and the control group. Each chicken group’s caecal microbiome and metabolome were characterized using 16S rRNA sequencing and Liquid chromatography coupled with mass spectrometry (LC–MS) analyses.

**Results and discussion:**

Our results showed that the on day 21, the final body weight was significantly reduced in response to OTA treatments (*p* < 0.05), the average daily gain in the OTA group was found to be inferior to the other groups (*p* < 0.01). In addition, Nano-Se supplementation could reduce the jejunum and liver pathological injuries caused by OTA exposure. The 16S rRNA sequencing suggest that Nano-Se supplementation in OTA-exposed chickens mitigated gut microbiota imbalances by promoting beneficial microbiota and suppressing detrimental bacteria. Moreover, untargeted metabolomics revealed a significant difference in caecal metabolites by Nano-Se pretreatment. Collectively, the dataset outcomes highlighted that Nano-Se augmentation regulates intestinal microbiota and associated metabolite profiles, thus influencing critical metabolic pathways, and points to a possible food-additive product.

## 1. Background

Ochratoxin A (OTA) is produced by various species of Penicillium and Aspergillus fungi (1). OTA is frequently found in various food sources such as coffee, beer, wine, fruits, vegetables, eggs, meat, animal feeds, and grains (2, 3). OTA has a broad distribution and high toxicity, leading to substantial financial losses within livestock and poultry industries. This situation poses challenges in meeting globalized meat and dairy product requirements and compromises food safety. Selenium (Se), an essential mineral for animal health, has garnered considerable interest in recent years (4–6). Organic forms of Se (such as selenomethionine, selenocysteine, and Se-enriched yeast), inorganic forms (such as sodium selenite), and nano forms are currently utilized as poultry feed additives (7). In previous studies, nano-Se has demonstrated superior bioavailability to its inorganic counterparts (8, 9). The intestinal microbiota, an essential constituent of the intestinal barrier, performs a critical function in preserving intestinal homeostasis and safeguarding intestinal tract health (10). The level of diversity exhibited by the gut microbiota indicates the intricate array of species present within it. Various external variables, including dietary intake, medications, and toxins derived from environmental sources, can affect gut microbiota composition. Research has demonstrated that mycotoxins, including but not limited to fumonisin B1, Zearalenone, aflatoxin B1, and Ochratoxin A, can potentially disrupt the balance of gut microbiota by reducing the population of beneficial bacteria and promoting the growth of pathogenic bacteria (11–13). Metabolomics, which analyzes and identifies metabolites in cells and tissues, is essential for disease diagnosis, drug development, and toxicology analysis (14–16). However, limited research exists on the mechanisms of action of mycotoxins like OTA based on metabolomics. This study investigated the impact of Nano-Se supplementation in diet on gut microbiota in OTA-challenged chickens using 16S rRNA analysis. LC–MS provides comprehensive coverage of substances and is considered the most stable and appropriate technique for studying metabolism. The cecal content was examined using an LC–MS metabolomics research strategy. The study screened endogenous metabolites between groups based on bioinformatics and analyzed metabolic pathways to identify potential biomarkers. This approach helped us understand the mechanism of Nano-Se action in OTA-exposed chickens.

## 2. Methodology

### 2.1. Materials

The OTA benchmarks utilized were obtained from Pribolab (Immunos, Singapore) and had a purity level exceeding 98%. The specimen of Nano-Se used in this study had a diameter ranging from 30 to 60 nm and a Se purity of 99.5%. The source for Nano-Se was the Bosar Biology cohort (Guangzhou, China). OTA crystals of high purity were solubilized in absolute ethanol at 1 mg per 10 mL. A suspension was created by mixing the resultant solution with 90 mL of sterile sunflower oil. Analytical-grade reagents were used in the experiment.

### 2.2. Animals

A cohort of 80 neonatal broiler chicks was procured from a commercial hatchery and allowed 3 days for acclimation after transportation to a novel environment. The chickens were provided with unrestricted access to both food and water. The formulation and nutritional content of the basal diet were designed in accordance with the NRC Nutrient Requirement (2012), as detailed in Table 1. The diet for each cohort was prepared concurrently and preserved in airtight containers prior to feeding, and the experiment lasted for 21 days.

**Table 1:** Composition and nutrient levels for basal experimental diet (%).

Item	Content (%)
Ingredients	
Corn	54.65
soybean meal	38.15
Soybean oil	2.62
Dicalcium phosphate	2.02
Limestone	1.18
Sodium chloride	0.22
Lysine	0.03
DL-methionine (Met)	0.13
Premix^a^	1.00
Total	100.00
Nutrient levels	
Metabolizable energy (ME)	12.54
Crude protein (CP)	21.15
Available phosphorus (P)	0.45
Calcium (Ca)	1.00
Lysine	1.10
Methionine	0.55

^a^Generally, in animal experiments, the metabolizable energy values of diets are calculated values. Supplied the following per kilogram of feed: vitamin A, 5000 IU; vitamin B2, 15 mg; pyridoxine, 4 mg; vitamin B1, 2.5 mg; vitamin D, 80.80 mg; vitamin E, 30 mg; pantothenic acid, 55 mg; niacin, 20 mg; biotin, 0.35 mg; folic acid, 1.55 mg; choline, 550 mg; Fe, 75 mg; Cu, 8.5 mg; Mn, 80 mg; I, 1.0 mg; Se, 0.07 mg; Zn, 65 mg; 116 mg.

### 2.3. Experimental design and treatment

The chicks were divided randomly into four equal groups, including the Se group (1 mg/kg Nano-Se in diet), the OTA group (50 μg/kg OTA by body weight), the OTA-Se group (50 μg/kg OTA + 1 mg/kg Nano-Se), and a control group. The dosages were recalibrated in alignment with the weight variations in the broilers (17, 18). Upon the experiment’s conclusion, all chickens were euthanized via cervical vessel bisection. Six broiler chickens from each group had their cecal contents frozen using sterile methods, and their liver and jejunum were fixed in 4% (w/v) paraformaldehyde. These tissues were subsequently processed through dehydration, infiltration, embedding, slicing, and staining with hematoxylin and eosin (H&E) in adherence to standard histopathological examination procedures.

### 2.4. 16S rRNA sequencing and bioinformatics analyses

QIAamp DNA^®^ Stool Mini Kit (Qiagen™, Germany) extracted enteric bacterial DNA from stored cecal contents. The 16S rDNA’s V3-V4 region was subjected to PCR amplification through primers 338F (ACTCCTACGGGAGGCAGCA) and 806R (GGACTACHVGGGTWTCTAAT). Amplicons were then evaluated through 2% gel electrophoresis. After that, AxyPrep DNA Gel Extraction^®^ (Axygen™, United States) retrieved tested amplicons from such gel. The detection and quantification of PCR products were carried out using a QuantiFluor™-ST micro fluorometer (Promega, Madison, WI, United States). Subsequently, MiSeq libraries were prepared for pyrosequencing upon MiSeq^®^ (Illumina™, United States). Sequences that exhibited a similarity of at least 97% were grouped into a single operational taxonomic unit (OTU) after being subjected to cleaning. The bioinformatics analyses were done through Majorbio^1^ (Shanghai, China). The platform was accessed on October 25, 2022. The study employed Partial Least Squares Discriminant Analysis (PLS-DA), demonstrating β-diversity analyses at the OUT level, utilizing weighted UniFrac indices and Bray-Curtis. Kruskal-Wallis rank sum test probed variations with statistical significance relating to abundance within phylum/genus levels.

### 2.5. Metabolome specimen processing

A 50 mg specimen of cecal content was precisely weighed and combined with 6 mm grinding beads, 400 mL extracting solution consisting of a 4:1 ratio of methanol to water (v:v), and a standard solution carrying 0.02 mg mL^−1^ standard (L-2-chlorophenylalanine). The resulting mixture was placed in a 2 mL centrifuging-tube. Following 6 min of tissue-grinding (−10°C/50 Hz), ultrasonic collecting was conducted (30 min/5°C/40 kHz). Subsequently, the specimen was subjected to a temperature of −20°C for 30 min and underwent centrifugation for 15 min at 9,400×*g* at 4°C. A volume of 20 μL (supernatant) from each specimen was transferred using a pipette into a specimen vial that contained an inner cannula for computerized assessment. One quality control (QC) specimen injection was interspersed after every six specimens. The specimens underwent analysis via LC–MS, with six biological replicates per group.

### 2.6. LC–MS detection of metabolites

LC–MS analysis was conducted using the UHPLC-Q Exactive^®^ platform (ThermoFisher-Scientific™, China). Chromatography parameters were set as follows: the column used was HSS T3 with dimensions of 100 mm × 2.1 mm inner diameter and a particle size of 1.8 μm. Mobile phase A consisted of 5% acetonitrile and 95% water with 0.1% formic acid, and mobile phase B contained 47.5% isopropyl alcohol, 47.5% acetonitrile, and 5% water with 0.1% formic acid. The volumetric flow rate was 0.40 mL/min, the injection volume was 2 μL, and the column temperature was 40°C. MS technique utilized electrospray ionization to obtain mass spectrum signals for specimens. The mass scanning range was set to 70–1,050 m/z, and both negative and positive ion scanning modes were employed. The experimental conditions encompassed the following operational parameters: a negative ion voltage of 2,800 V, positive ion voltage of 3,500 V, sheath gas pressure at 40 psi, auxiliary heating gas pressure at 10 psi, ion source heating temperature at 400°C, cyclic collision energy of 20-40-60 V, a primary mass spectrometry(MS1) resolution of 70,000, together with secondary mass spectrometry (MS2) resolution of 17,500.

### 2.7. Data preprocessing and database searching

Progenesis QI software processed raw data, standardizing specimen response intensities from mass spectrum peaks to minimize errors arising from specimen preparation and instrument instability. Specimens with a relative standard deviation (RSD) > 30% were excluded from assessments. The eventual data matrix for further investigation was obtained by performing a logarithmic transformation on the variables. Metabolomic data were acquired by integrating MS1 and MS2 spectra through publicly available metabolic database datasets, namely HMDB^2^ and Metlin.^3^

### 2.8. Orthogonal projections to latent structures discriminant analysis (OPLS-DA)

OPLS-DA gained insight into metabolic variations both within and between specimen groups. Metabolites that exhibited significant differences were chosen depending upon their Variable Important in Projection (VIP) scores and *p*-values obtained from Student’s *t*-test. Metabolites exhibiting a VIP score of ≥1 and *p* < 0.05 were assumed to have statistical significance. Subsequently, differential metabolites were identified and classified through the Kyoto Encyclopedia of Genes and Genomes (KEGG)^4^ to determine the impacted metabolic pathways. KEGG pathways were deemed to be significantly enriched if they had *p* < 0.05.

### 2.9. Statistical analyses

Experimental results were reported in the form of mean values along with their corresponding standard deviations. The anticoccidial effects were analyzed using the One-way ANOVA test. The statistical methods for T-/Wilcoxon rank sum−/Tukey-tests evaluated disparities across all four treatments. *p* < 0.05 was deemed to confer statistical significance.

## 3. Results

### 3.1. Growth performance

Table 2 presents the impact of OTA and Nano-Se supplementation on the growth performance of broilers. There were no statistically significant differences (*p* > 0.10) observed in the initial body weight (IBW) among the groups. However, on day 21, the final body weight (FBW) was significantly reduced in response to OTA treatments (*p* < 0.05). Additionally, the average daily gain (ADG) in the OTA group was found to be inferior to the other groups (*p* < 0.01). However, the average daily feed intake (ADFI) remained consistent across all four groups. Pertaining to the feed conversion ratio (FCR), a decrease in FCR was noted with the addition of Nano-Se from day 1 to 21 (*p* < 0.05).

**Table 2:** The effect of OTA and Nano-Se supplementation on growth performance of broilers from 1 to 21 d.

Treatment	CON	OTA	OTA-Se	Se	*p*-value
IBW (g)	39.25 ± 0.42	39.23 ± 0.48	39.38 ± 0.37	39.31 ± 0.33	0.988
FBW (g)	616.32 ± 10.36^a^	503.21 ± 14.25^b^	608.22 ± 12.55^a^	614.95 ± 11.96^a^	0.027
ADG (g/d)	27.43 ± 0.55^a^	22.11 ± 0.35^b^	27.25 ± 0.42^a^	29.07 ± 0.32^a^	0.023
ADFI	41.0.56 ± 0.42	41.94 ± 0.35	42.0.63 ± 0.55	42.43 ± 0.77	0.812
FCR	1.52 ± 0.03^a^	1.87 ± 0.01^b^	1.56 ± 0.02^a^	1.46 ± 0.01^c^	0.015

CON, a basal diet; Se, a basal diet + 1 mg/kg Nano-Se; OTA, a basal diet + 50 μg / kg OTA; OTA-Se, a basal diet + 50 μg / kg OTA + 1 mg/kg Nano-Se; IBW, initial body weight; FBW, final body weight; ADG, average daily gain; ADFI, average daily feed intake; FCR, feed conversion ratio. Data were shown as Mean ± SEM and analyzed by one-way ANOVA Tukey’s test. Different letters (a, b, c) in each parameter represent significance (*p* < 0.05).

### 3.2. Histopathological changes in the intestinal and liver

**Figure 1:**
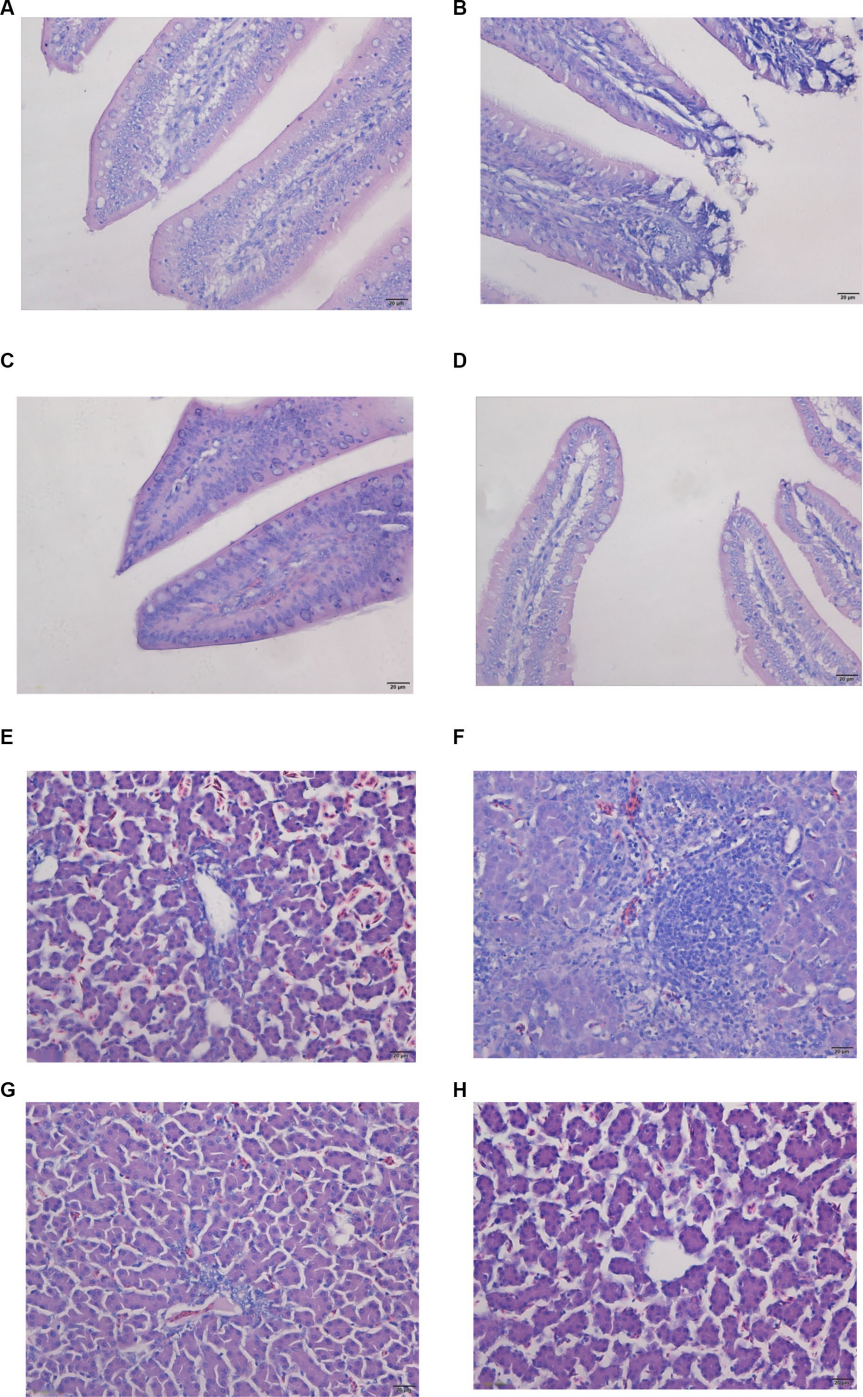
Pathological changes in the jejunum and liver were detected by tissue section H&E staining. Images were taken at magnifications of 400×. **(A,E)** Control group; **(B,F)** OTA group; **(C,G)** OTA-Se group; **(D,H)** Se group.

The jejunum’s morphological features were normal in both the control and the Se treatment groups, exhibiting a complete intestinal mucous membrane structure. The intestinal villi were clear and well-aligned, without significant abnormal changes (Figures 1A,D). Conversely, the jejunum of broilers in the OTA group was notably distended, with extensive detachment of the intestinal mucosa and observable structural damage (Figure 1B). Following pretreatment with Nano-Se, the detachment of the intestinal mucosa in the broiler jejunum was mitigated, and no discernible changes were seen in the lamina propria (Figure 1C).

HE staining further clarified the condition of the liver tissue. In the chickens from the control and Se groups, no signs of inflammation, congestion, bleeding, necrosis, or exudation were observed (Figures 1E,H). In the OTA group, the liver displayed signs of inflammation, most notably due to OTA-triggered extensive infiltration of inflammatory cells, particularly a high concentration of lymphocytes and eosinophils (Figure 1F). Minimal pathomorphological alterations were detected in the OTA-Se group (Figure 1G). Collectively, these findings indicate that Nano-Se supplementation provided a protective effect against OTA-induced hepatic injury in chickens.

### 3.3. DNA sequence data

**Figure 2:**
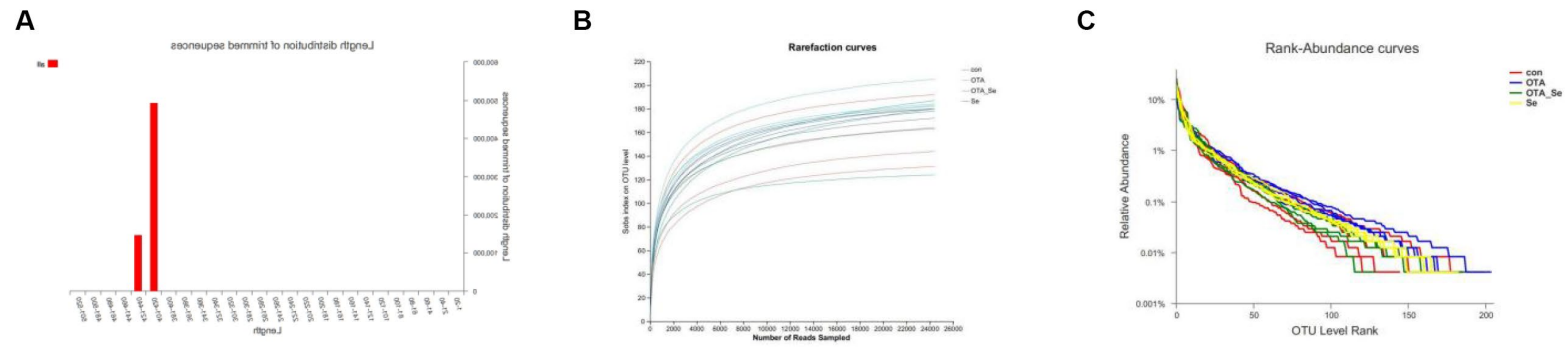
**(A)** Fragment length distribution of sequences from each sample after merging and trimming. **(B)** The rarefaction curves plotted to visualize the OTUs of each group. The red line represents the control group, the blue line the OTA group, the green line the OTA-Se group, and the purple line the Se group. **(C)** Rank abundance curves of gut microbiota at the OUT level in the four groups.

High-throughput sequencing analysis for chicken cecal contents was conducted using the Illumina MiSeq platform. After QC, 640,107 valid sequences were obtained, averaging 40,006 sequences per specimen. Among these high-quality sequences, approximately 99.95% were longer than 400 bp, with the majority ranging between 401 and 420 bp (Figure 2A).

Based on a 97% similarity threshold, all effective reads were grouped into OTUs. A plateau was reached by the rarefaction curves for the four groups, suggesting that sequencing depth employed within such an investigation sufficed for assessing specimen-derived microbial population makeups and accurately depicting faecal microbial colonies (Figure 2B). The microbiota composition’s richness and evenness were comparable among the four groups by the rank abundance analysis within the OTU level (Figure 2C). The adequacy of sampling efforts was supported by both rarefaction and rank abundance curves for each specimen.

### 3.4. Effects of Ochratoxin A and Nano-Se on the gut microbial composition

Alpha diversity measures the number of species and their diversity within a particular specimen. Alpha diversity can be evaluated using different indices. The indices mentioned above comprise ACE, Chao1, Simpson, and Shannon. The ACE and Chao1 indices quantify the abundance of species, which refers to overall species quantities present within a given specimen. On the other hand, the Simpson and Shannon indices assess species’ diversity, considering the number of species and their relative abundance within the specimen. The alpha diversity analysis indicated that there were no statistically significant variations within the Simpson index, Shannon index, Chao1 index, and ACE index across all four therapeutic cohorts (*p* > 0.05) (Table 3).

**Table 3:** Microbial diversity indices in different treatment groups at d 21.

Items	CON	CON-SD	OTA	OTA-SD	OTA-Se	OTA_Se-SD	Se	Se-SD	*p*-value
Simpson	0.06	0.02	0.04	0.01	0.06	0.01	0.05	0.01	0.885
Chao1	165.41	28.43	199.93	10.39	179.73	40.22	185.29	5.44	0.312
ACE	165.72	27.47	194.21	10.91	179.72	37.12	186.87	4.21	0.311
Shannon	3.37	0.38	3.72	0.14	3.39	0.11	3.51	0.03	0.665

CON, a basal diet; Se, a basal diet + 1 mg/kg Nano-Se; OTA, a basal diet + 50 μg / kg OTA; OTA-Se, a basal diet + 50 μg / kg OTA + 1 mg/kg Nano-Se; The ACE index and Chao1 index represent the community richness of the microbiota, and the Shannon and Simpson indexes represent the community diversity of the microbiota; SD, standard error of the mean.

**Figure 3:**
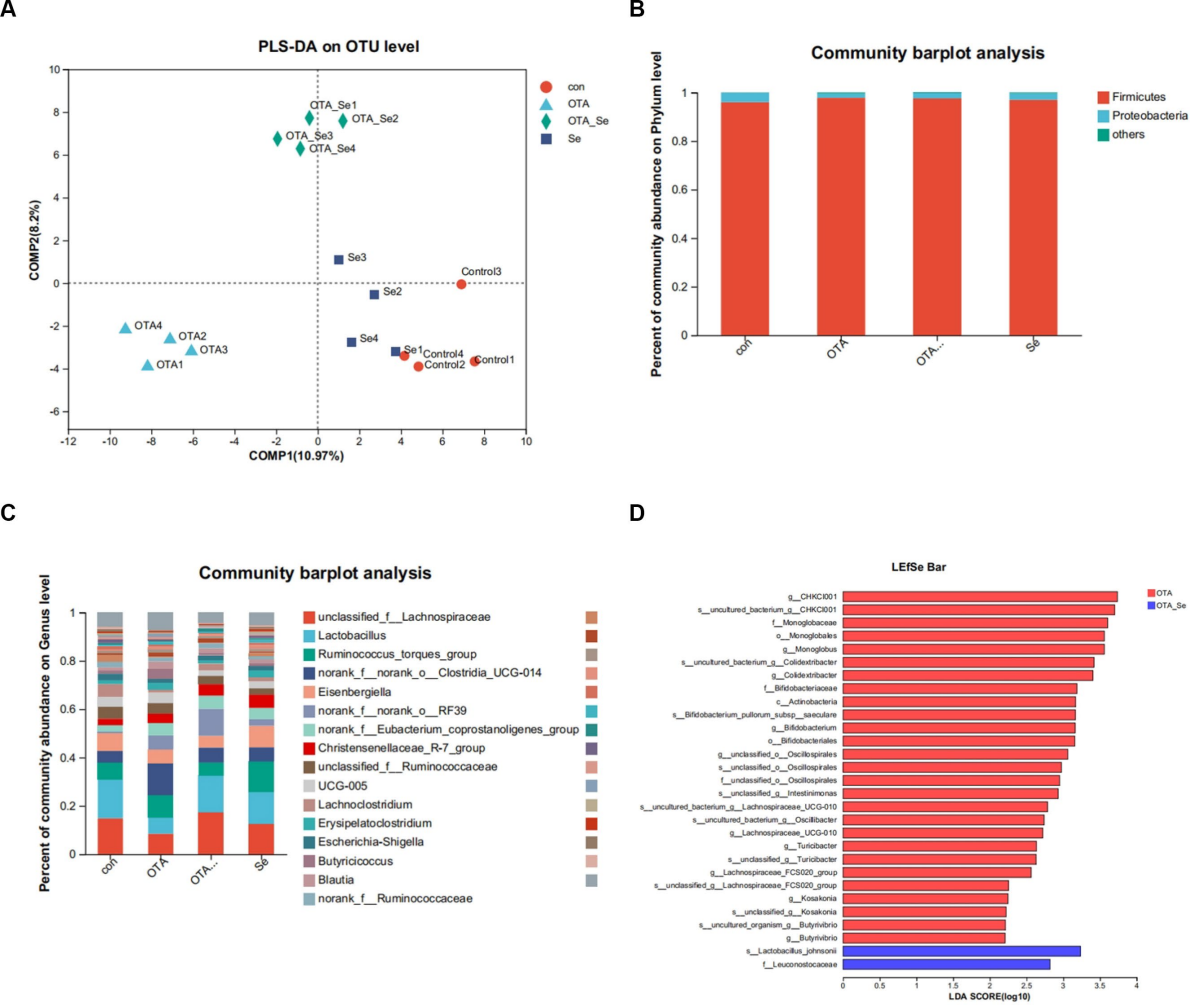
**(A)** The impact of OTA and Nano-Se on the structure of the microbial community in the cecum of chickens evaluated through PLS-DA. Each data point represents an individual sample, and data points that share the same colour belong to the same group. A superior classification model is suggested when the intra-group distance is minimized and the inter-group distance is maximized. **(B)** Relative abundance of phyla in gut microbiota. **(C)** Relative abundance of genera in gut microbiota. **(D)** LEfSe bar based on phylum to genus level (LDA > 2). *p* < 0.05.

Beta diversity assessed variations within species complexity within specimens, as demonstrated by a weighted Unifrac index. Regarding β-diversity, PLS-DA revealed that the similarity in species diversity significantly differed when chickens were given OTA and Nano-Se (Figure 3A). A distinct separation between the groups was observed within the chicken’s cecal flora, indicating that the structure of the chicken’s cecal flora had been altered.

As illustrated in Figure 3B, the primary phylum of broiler gut microbiota was *Firmicutes*, followed by *Proteobacteria*. Relative abundance for *Firmicutes* within the OTA cohort was higher than the control cohort and similar to the OTA-Se group.

The predominant genera within the gut included *Lactobacillus*, *norank_f__norank_o__Clostridia_UCG-014, unclassified_f__Lachnospiraceae*, and *Ruminococcus_torques_group* (Figure 3C). Among these genera, the relative abundance of *Lachnospiraceae* was highest within the OTA-Se group. Conversely, the relative abundance of *Clostridia* was highest within the OTA group.

A supervised specimens comparison was conducted using LEfSe and logarithmic linear discriminant analysis (LDA). The LEfSe results, displayed in Figure 3D, revealed that compared to the control group, the dominant bacteria within the OTA-Se cohort were *s__Lactobacillus_johnsonii* and *f__Leuconostocaceae* at phylum to genus levels.

### 3.5. Metabolome analysis

Quality control specimens assessed consistency/reproducibility for investigational methods, with arbitrary injections performed during specimen injection. More than 70% of RSD values for the QC specimens were less than 30%, suggesting datasets collected through such investigational methods exhibited stability.

**Figure 4:**
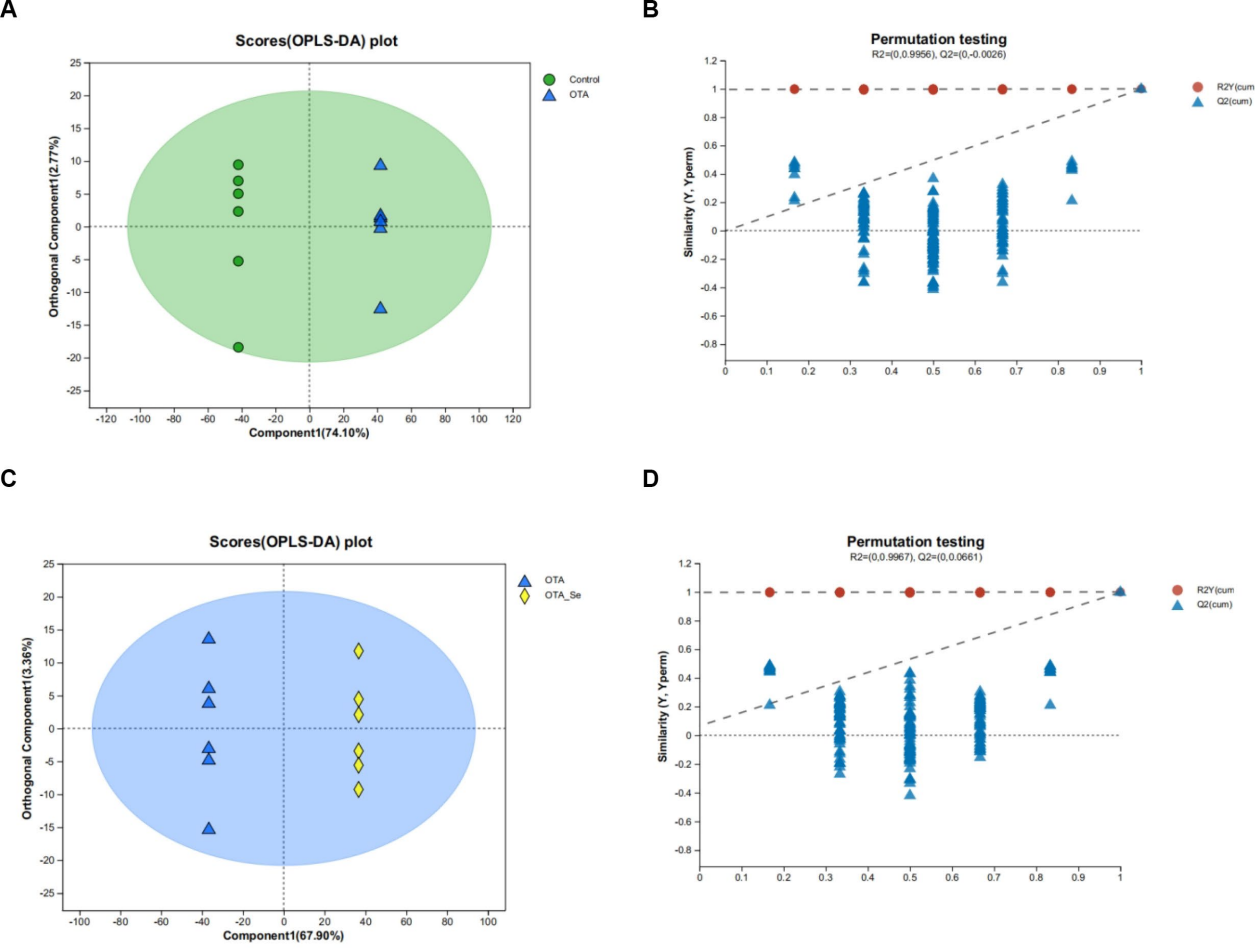
OPLS-DA model plot for the comparison group Control *vs* OTA: **(A)** score diagram; **(B)** model validation diagram; OPLS-DA model plot for the comparison group OTA *vs* OTA-Se; **(C)** score diagram; **(D)** model validation diagram.

To explore the changes in gut metabolites induced by dietary OTA and Nano-Se supplementation, an untargeted metabolomics approach was applied to analyze the metabolite profiles of the cecal content. OPLS-DA was utilized to discern distinct metabolic patterns, as illustrated in Figures 4A,C. This analysis revealed separate metabolic profiles between the OTA and Control groups and between the OTA-Se and OTA cohorts. OPLS-DA allowed for filtering irrelevant variations, enhancing the ability to identify substantial differential metabolites among the different groups. The closeness of duplicate points for the two sample groups indicates good data repeatability.

OPLS-DA model validation was carried out within this investigation to avoid overfitting. Clear separations between OTA (blue dots) and the Control control (green dots) groups (R2 = 0.9956, Q2 = −0.0026, Figure 4B) and between OTA (blue dots) and OTA-Se (yellow dots) groups (R2 = 0.9967, Q2 = 0.0661, Figure 4D) were demonstrated by the OPLS-DA models. The higher the R2 and Q2 values, the greater the theoretical reliability for this model. The acquired data suggest that both models demonstrate favourable stability and no overfitting. The material is confirmed to be sufficiently reproducible and appropriate for subsequent qualitative and quantitative assay validations, as demonstrated by these results.

### 3.6. Differential metabolite analysis

**Figure 5:**
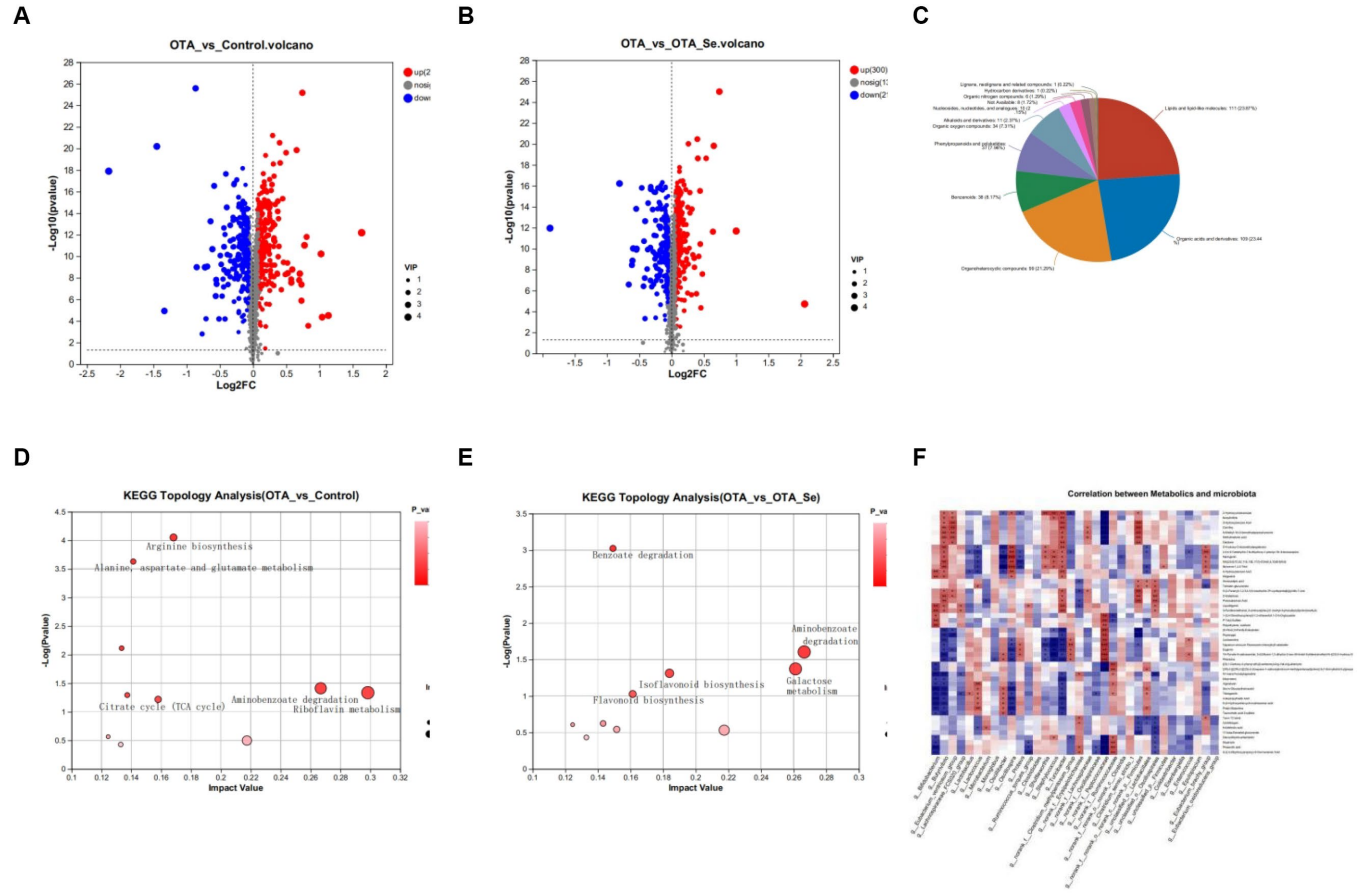
**(A)** Differential metabolite expression analysis to compare the control group with the OTA group. **(B)** The volcano plots exhibited distinct metabolites in the OTA-Se group compared to the OTA group. The red colour is indicative of an elevation in the content of metabolites, while blue signifies a reduction in metabolite content. The grey colour represents the absence of any noteworthy difference. **(C)** Classification of differential metabolites in the HMDB database. **(D)** Bubble diagram showing the KEGG enrichment analysis. Con *vs* OTA. **(E)** The KEGG enrichment analysis OTA *vs* OTA-Se. **(F)** Spearman’s rank correlation between cecum metabolites and associated gut microbiota in OTA and OTA-Se group. Connections depicted in red signify a direct positive correlation, whereas those in blue represent an inverse negative correlation. The statistical significance of the data is calculated using a two-tailed Wilcoxon rank-sum test to determine the *p*-values. *p < 0.05, **p < 0.01.

Metabolites were selected differentially by utilizing VIP value (VIP > 1) from the OPLS-DA model together with *p* value (*p* < 0.05) from stand-alone specimen *t*-test from all identified metabolites. The chosen differential metabolites served as marker variables to distinguish across cohorts. Compared to the control cohort, the OTA cohort exhibited an initial identification of 515 differential metabolites, with 259 within positive mode and 256 within negative ion mode. Among these metabolites, 276 were up-regulated, while 239 were downregulated (Figure 5A). Upon comparison for OTA and OTA-Se groups, a collective of 514 distinct metabolites (277 within positive mode and 237 within negative ion mode) were detected through both modes of analysis (Figure 5B). Among the recognized metabolites, 214 displayed a significant decrease, while 300 exhibited an increase.

The differential metabolites identified in this study were annotated within HMDB to be classified. The results showed that lipids/lipid-like molecules constituted 23.87% of differentially aggregated metabolites within OTA and OTA-Se cohorts. Organic acids/derivatives were the second most abundant cohort at 23.44%, followed by organoheterocyclic compounds at 21.29%, benzenoids at 8.17%, phenylpropanoids, and polyketides at 7.96%, organic oxygen compounds at 7.31%, alkaloids and derivatives at 2.37%, nucleosides, nucleotides, and analogues at 2.15%, organic nitrogen compounds at 1.29%, and the remaining compounds at 0.44% (Figure 5C).

Further analysis of identified metabolites was conducted using the KEGG analysis to investigate potential pathways impacted by OTA and Nano-Se interventions. The top 10 KEGG metabolic pathways with significant enrichment for different metabolites were found using the KEGG database. Compared to the control cohort, pathways with the highest impact values within the OTA cohort included Riboflavin metabolism, Aminobenzoate degradation, Limonene and pinene degradation, Arginine biosynthesis, and Alanine, aspartate, and glutamate metabolism. Among the metabolites implicated within these pathways, Riboflavin, Benzoate, Oxoglutaric acid, and L-Glutamine were notably decreased within the OTA cohort compared to the control group. Conversely, Benzoic Acid, Gallic Acid, and Citrulline increased within the OTA cohort relative to the control cohort (Figure 5D).

When comparing the OTA cohort with the OTA-Se cohort, the distinct metabolic pathways within intestinal contents included Aminobenzoate degradation, Galactose metabolism, Isoflavonoid biosynthesis, and Flavonoid biosynthesis. Among the metabolites participating in these metabolic pathways, Protocatechuic Acid, 4-Hydroxybenzoic Acid, D-Galactose, Daidzein, Liquiritigenin, and Naringenin were highly raised within the OTA-Se cohort in comparison with the OTA group. Conversely, Galactonic acid and Myo-Inositol were reduced within the OTA-Se cohort compared to the OTA cohort (Figure 5E).

The correlation between different microbial genera and the modified cecal metabolites was determined using Spearman’s correlation coefficient values in a correlation analysis (Figure 5F). The present study has revealed associations having considerable statistical significance (*p* < 0.05, *r* > 0.70) across particular bacteria present within the cecum and distinctive metabolites in comparing OTA *vs* OTA-Se. Two distinct metabolites, Daidzein and Liquiritigenin, involved within the Isoflavonoid biosynthesis pathway, exhibited significant positive correlations with Firmicutes, Turicibacter, and Butyrivibrio. Furthermore, Protocatechuic Acid, present within Aminobenzoate degradation and Benzoate degradation pathways, demonstrated a positive correlation with *Oscillospirales*, *Firmicutes*, and *Butyrivibrio* while revealing a negative correlation with *Erysipelotrichaceae*. Although the direct metabolism of these metabolic products by gut microbiota remains conclusively determined, the results highlight a close interplay between gut microbial species and metabolites, suggesting that Nano-Se supplementation may have induced notable alterations in gut microbiota, ultimately leading to significant shifts in host metabolite abundance.

## 4. Discussion

OTA (C20H18ClNO6; molecular weight:403.8) is a predominant mycotoxin originating from various Penicillium and Aspergillus species. Its pervasive presence and contamination of crops and foodstuffs pose significant health threats to humans and animals (2). Se, an essential trace element for mammals, including humans, exhibits a strong correlation between its physiological functions and disease prevalence (19). Previous research has highlighted Se′s crucial role in diminishing the damage inflicted on animals by mycotoxins (20–22). In the current investigation, chickens fed with OTA-contaminated feed displayed a marked decrease in FBW, ADG and a notable elevation in FCR findings congruent with prior studies (23, 24). Additionally, existing literature has demonstrated that even minor exposure to OTA in animals can provoke pathological and functional alterations in the liver and intestines (25–30). Our findings align with these earlier studies, indicating that Nano-Se supplementation can protect chickens against OTA-induced harm. Gut microbiota, recognized as a critical and responsive indicator of gastrointestinal health, is inevitably influenced by environmental contaminants, including mycotoxins such as OTA. In recent years, a growing body of evidence has demonstrated that OTA has a detrimental effect on the integrity of the intestinal barrier, induces oxidative stress and inflammation, and lowers the abundance of beneficial microorganisms (31–35). OTA induces alterations within gut microbiota composition, affecting the phylum, genus, and species levels (36). Literature has documented a considerable increase in Bacteroides enrichment in mice exposed to OTA (37). Research on broilers and ducks has indicated that OTA lowers abundance/diversity for cecal microbiota, triggering intestinal tight junction damage (38).

Recently, the scientific community has exhibited increased curiosity in understanding the modulatory effects of Se on the intestinal microbiota. When exposed to selenium-enriched yeast, broilers subjected to OTA experienced alterations in the diversity of their caecal microbiota (30). Zhang et al., while analyzing variations in Se distribution in the Enshi region of Hubei, China, noted a heightened prevalence of Bacteroidetes in areas with higher Se concentrations as opposed to regions with lower Se concentrations (39). Their findings revealed that dietary Se supplementation can influence the gut’s immunological reactions and barrier functionality. This is linked to the alteration of gut microbiota via faecal microbiota transplantation procedures (40). Furthermore, dietary Se supplementation noticeably enhanced mice microbiota diversity (41). Yeast Se and selenium-enriched yeast culture supplementation contributed to intestinal homeostasis by elevating the relative abundance of anti-inflammatory-associated microbiota, thereby mitigating damage caused by *S. enteritidis* infection (42). The research findings indicate that adding dietary Se Nanoparticles (SeNPs) can effectively modulate the gut microbiota and its metabolic processes, reducing the severity of acute toxicity caused by diquat (43). Consuming SeNPs exceeding nutritional requirements may enhance gut microbiota composition, thereby protecting against intestinal dysfunctions (44). An intermediate concentration of SeNPs at 0.9 mg/kg demonstrated the highest efficacy in enhancing gut health by promoting beneficial bacteria, including *Faecalibacterium* and *Lactobacillus* (45).

The present study determined that the *Clostridia* populations increased while *Lactobacillus* and *Lachnospiraceae* abundance decreased within the OTA group, but these levels normalized following Nano-Se supplementation. *Clostridium*, including species like *Clostridium perfringens*, *Clostridium botulinum*, and *Clostridium tetani*, can generate exotoxins and are associated with numerous diseases (46). Research has revealed a positive correlation between *Clostridium* and monocyte chemoattractant protein-1 (MCP-1), a pro-inflammatory cytokine (47). The level of Clostridium bacteria was found to be strongly correlated with the severity of inflammatory bowel disease (IBD) (48) within the gut microbiota of chickens exposed to OTA, oral administration of aflatoxin B1 (AFB1) diminished the alpha diversity of gut microbiota in broilers and augmented the abundance of several detrimental bacteria, including *Clostridium* (49). In addition, AFB1 significantly increased *Bacteroidales* of *Bacteroidetes* and *Clostridiales* of *Firmicutes* in rats, while *Lactococcus* sp., *Streptococcus* sp., and *Lactobacillales* from *Firmicutes* decreased (50). Another study found that Deoxynivalenol (DON)treatment increased the abundance of *Clostridiales* within gut microbiota (51, 52). In other studies, the OTA cohort had significantly higher levels of *Aerococcus, Romboutsia*, and *Clostridium sensu stricto 1*. However, Tibetan kefir reduced the abundance of these three bacteria (53). These findings imply that mycotoxin alters intestinal microbiota makeup and encourages the growth of harmful bacteria, producing toxic effects.

Furthermore, *Lactobacillus* abundance increased considerably within the OTA-Se cohort compared to other groups. It is well-established that *Lactobacillus* has pivotal parts in keeping healthy together with preventing and treating diseases (54, 55). With numerous beneficial effects, *Lactobacillus* produces lactate, which can elevate butyrate production in faeces (56). *Lactobacillus johnsonii* has exhibited anti-obesity properties by inhibiting gut inflammation and maintaining the integrity of the mucosal barrier (57). The high-energy diet feeding cohort exhibited a greater abundance of Lactobacillaceae species, specifically *Lactobacillus reuteri* and *Lactobacillus johnsonii*, within the upper intestine (duodenum and cecum). This colonization led to protective effects on the mucosal lining and reduced inflammation (58). The investigation concerning OTA influence on intestinal microbiota revealed decreased levels of advantageous microorganisms such as *bifidobacteria* and *Lactobacillus*. This decrease indicates that OTA altered the balance of microbiota, which could result in compromised immunity (59). Mice administered with OTA through intragastric means exhibited a reduction within the proportionate prevalence of *Bifidobacterium* spp. and *Lactobacillus* spp. (60). Furthermore, Zearalenone elicited an increase in *Desulfovibrio* and a decrease in *Lactobacillus* abundance within the colon tissue of mice (61). The combination of *Bacillus cereus* BC7 and *Lactobacilli* strain positively impacted intestinal inflammatory responses and microbiota disturbances induced by Zearalenone (62, 63). The results indicated *Lactobacillus’s* increased resistance to OTA and its potential role within the OTA detoxification process.

The bacterial family, *Lachnospiraceae*, can convert lactate and acetate into butyrate. This conversion can be achieved using different enzymatic pathways, such as butyryl-CoA or acetate CoA transferase pathways or the butyrate kinase pathway (64) in addition to its butyrate production capabilities, *Lachnospiraceae* is known to participate in the biosynthesis of vitamin B12 and exhibit the potential to inhibit the colonization of *Clostridium difficile* within the gastrointestinal tract (65). The family *Lachnospiraceae* has been documented to have crucial functions in acquiring dietary glycans, synthesizing advantageous metabolites, enhancing immunity, and facilitating neurodevelopment in animals (66). Studies have shown that administering DON to pigs at a dose of 2.89 mg/kg resulted in various alterations within the cecal microbiota. This ushered a major decrease within levels of unclassified *f_Lachnospiraceae*, which was found to have a positive correlation with the average daily feed intake (67). The administration of OTA to mice, followed by the supplementation of Tibetan kefir, resulted in a significant increase in the abundance of bacterial populations that develop short-chain fatty acids (SCFAs), such as *Lachnospiraceae*, *Ruminococcus*, and *Blautia*. SCFAs are synthesized through intestinal microbiota via anaerobic fermentation. These compounds enhance the integrity of the intestinal barrier and suppress inflammatory reactions (68). Given the positive effects of Se on chickens’ gut microbiota composition, the current results suggest that Se could enhance gut health and serve as a candidate additive for promoting intestinal health in OTA-exposed chickens. However, further investigation is required to elucidate the mechanism through which Se promotes the growth of intestinal microorganisms.

Metabolomics represents a novel analytical approach for detecting changes in small endogenous metabolites. External factors or internal disruptions may influence these alterations, and metabolomics can be employed to diagnose and predict the underlying mechanisms of such changes (69, 70). Multiple research investigations have documented the impact of Se consumption on metabolic processes (43, 71, 72). 515 distinct metabolites within the cecum of broiler chickens were identified through metabolomics analysis after an OTA challenge. KEGG analysis revealed that these metabolites are enriched in riboflavin metabolism, aminobenzoate degradation, limonene and pinene degradation, arginine biosynthesis, and alanine, aspartate, and glutamate metabolism.

Riboflavin metabolism is intricately linked to the energy metabolism of mitochondria. Riboflavin is a precursor for two coenzymes, flavin adenine dinucleotide and flavin mononucleotide. These coenzymes are pivotal in several biological redox reactions, including electron transport chain, fatty acid oxidation, amino acid degradation, and tricarboxylic acid cycle (73–76). Numerous studies have demonstrated riboflavin’s ability to preserve the intestinal tract’s structural integrity and promote optimal gastrointestinal performance in various animal species (77). Riboflavin can regulate various pathways that are involved in the maintenance of gastrointestinal function. Riboflavin has been found to increase iron absorption and play a role within the antioxidant system. *In vitro* and *in vivo* investigations demonstrated that riboflavin deficiency can lead to oxidative stress within the intestines (78). Furthermore, studies have demonstrated that the level of riboflavin in an organism can affect the composition of its gut microbiome, and a riboflavin deficiency can lead to an imbalance within gut microflora (79–84). Hence, the diminished concentrations of riboflavin indicate that OTA could potentially induce harm to the intestines by altering riboflavin metabolism.

The anti-inflammatory properties of limonene and pinene have been extensively documented in scientific literature. Limonene, a naturally occurring monocyclic terpene hydrocarbon in citrus fruits, exhibits anti-inflammatory characteristics by selectively binding to A2A receptors and reducing inflammation (85). α-pinene, a bicyclic monoterpene hydrocarbon, is commonly present within essential oils of coniferous trees and is a major constituent of volatile organic compounds derived from diverse tree species. This compound exhibits a range of biological activities, including anti-inflammatory properties (86). Studies have demonstrated that α-pinene exhibits anti-inflammatory properties and holds potential as an alternative therapeutic intervention for inflammation (87). Based on the observed properties, it is a reasonable hypothesis that the increased activity of limonene and pinene degradation pathways within the OTA cohort could potentially reduce their concentrations, thereby mitigating the inflammatory reaction induced by OTA. However, additional investigation is required to comprehend the fundamental mechanisms.

L-Arginine is integral to numerous processes, including the ornithine cycle, protein synthesis, nitric oxide generation, oxidative stress management, and immune response regulation. Metabolomic alterations in cells exposed to ZEA revealed a significant enrichment of arginine biosynthesis-related differential metabolites. Subsequent research demonstrated that L-arginine supplementation considerably enhanced cell viability and diminished reactive oxygen species (ROS) production levels in Zearalenone-exposed cells (88). Moreover, OTA notably reduced the levels of differential metabolites, such as L-Glutamine, associated with arginine biosynthesis compared to the control group. L-Glutamine, a nonessential amino acid, is a critical energy source and an active free radical scavenger for enterocyte and lymphocyte cells. The intestine can utilize approximately 30% of total L-Glutamine, emphasizing its importance as a key nutrient for intestinal health (89). L-Glutamine has been shown to stimulate cell proliferation and differentiation, playing a crucial role in supporting the growth and development of the gastrointestinal tract (90). L-Glutamine supplementation has been confirmed to preserve intestinal tissue integrity and bolster the small intestine mucosa barrier (91). Both animal and clinical studies suggest that L-Glutamine offers protection against stress, pathogenic organism invasion, infection, and immunological challenges *in vitro* and *in vivo* (92, 93).

Based on these findings, it can be speculated that the downregulation of arginase-related pathways results in decreased arginine metabolism within the examined model. The outcomes indicate that OTA significantly impacts relevant metabolic pathways through its effects on metabolites, ultimately leading to detrimental consequences on nutrient metabolism and intestinal morphology.

KEGG analysis comparing OTA and OTA-Se groups revealed that Nano-Se could influence aminobenzoate and benzoate degradation by elevating 4-Hydroxybenzoic Acid, Protocatechuic Acid levels. This aminobenzoate degradation pathway may promote tryptophan metabolism and benzoate breakdown (94). Protocatechuic acid, a primary metabolite of complex polyphenols, exhibits numerous biological activities, encompassing antioxidant, anti-inflammatory, antibacterial, and antiapoptotic properties (95–97). Prior research has reported protocatechuic acid’s potential to attenuate intestinal damage (98). A study demonstrated that the inclusion of protocatechuic acid within yellow-feathered broilers’ diet significantly improved their growth performance, antioxidant capacity, gut immune function, and gut microbiota structure (99). Research utilizing piglets challenged with LPS revealed that protocatechuic acid mitigated oxidative stress, inflammation, intestinal barrier impairment, and gut flora disruption (100). Furthermore, protocatechuic acid demonstrates promise as a detoxifying agent against Fumonisin B1 and AFB1 (101, 102).

Additionally, upregulation of Daidzein, Liquiritigenin, and Naringenin within the OTA-Se group was observed, enhancing Isoflavonoid biosynthesis and Flavonoid biosynthesis. Flavonoids are recognized for their antioxidant and anti-inflammatory properties (103). Certain flavonoids, such as naringin and luteolin, have been identified to alleviate intestinal inflammation (104, 105). It has been reported that plant-derived flavonoids can modulate intestinal flora composition and maintain intestinal and organism health (106). Daidzein, recognized as a safe and natural alternative estrogen-like compound, has been increasingly investigated in scientific research. Studies conducted in avian science have revealed that daidzein exhibits potential as a dietary supplement for augmenting reproductive organ development, eggshell quality, and laying performance in laying hens during the final stages of the laying cycle (107, 108). The Daidzein and CH mixture significantly enhanced SOD and GSH-px activity in plasma (109). *Slackia* sp., D-G6, a bacterium capable of detoxifying deoxynivalenol, was discovered within the intestinal tract of chickens. Besides deoxynivalenol detoxification, the D-G6 enzyme also catalyzes the conversion of daidzein to equol. This compound exhibits potent estrogenic effects and effectively mitigates the risk of estrogen-dependent and age-related ailments (110). The flavonoid Liquiritigenin exhibits antiapoptotic, anti-inflammatory, antioxidant, and anti-fibrotic pharmacological properties (111). Naringenin, a naturally occurring flavonoid, exhibits antioxidative, antiproliferative, anti-inflammatory, and antimutagenic characteristics (112). Previous studies have demonstrated that naringenin provides cellular protection against oxidative damage induced by arsenic (113).

The gastrointestinal tract serves as the primary location of exposure to OTA and is pivotal for OTA-induced toxicity. The primary manifestation of OTA’s enteric toxicity is the induction of oxidative stress and inflammatory response, and the impact of OTA intoxication on gene expression of signalling pathway markers related to inflammation and inflammatory cytokines was more pronounced within the gut compared to the kidney of piglets (114). Research has demonstrated that OTA causes oxidative damage to the intestinal tract of broiler chickens, leading to pro-inflammatory cytokines elevation and anti-inflammatory cytokines reduction within the cecal tissues of these birds (40, 115). Studies conducted *in vitro* and *in vivo* have shown that OTA exposure leads to an overproduction of free radicals, which disrupts the equilibrium between antioxidant and oxidant systems and impairs the function and structure of cell membranes (11). The findings indicate that subchronic exposure to a low dosage of OTA for 30 days significantly impacts the immune response and antioxidant self-defence mechanisms within the gut and kidney. Furthermore, there have been reports linking disruption of intestinal microbiota to intestinal damage, inflammation, and compromised barrier integrity (116). As a result, the elevation of these anti-inflammatory and antioxidant metabolites(Protocatechuic Acid, Daidzein, Liquiritigenin, and Naringenin) due to Se intervention may bolster the anti-inflammatory and antioxidant capabilities of host tissue. Se supplementation in OTA-exposed chickens can safeguard the intestine by modulating metabolites. Future studies should further verify these findings and delve into the underlying mechanism of Se in enhancing gut health more comprehensively using various experimental models. Moreover, the relationship between gut microbiota and the gut microbiome warrants further exploration.

## 5. Conclusion

In summary, this investigation probed the protective influence of Nano-Se in OTA-fed chickens. Results from the analysis of 16S rRNA sequencing and untargeted metabolomics indicated that Nano-Se could modulate the intestinal microbiota community and metabolites. Supplementation of Nano-Se has the potential to mitigate alterations in gut microbiota caused by OTA exposure through the promotion of favourable microbial populations and suppression of pathogenic bacteria. Furthermore, Nano-Se treatment also influenced the contents of cecal metabolites. This work offers a novel approach to investigating the protective role of Nano-Se within enterotoxicity induced by OTA in chickens. To the best of our knowledge, this is the first research exploring OTA’s toxic impact on poultry, especially concerning gut microbiota and intestinal metabolic responses. Our results contribute to a deeper understanding of how gut microbiota is involved in OTA-induced intestinal barrier disruptions and suggest potential innovative preventive measures against such dysfunctions. Notably, our investigation was limited to the effects of a single mycotoxin on chickens, even though myriad mycotoxins are prevalent in livestock farming. Therefore, more comprehensive studies are warranted to elucidate the impacts of diverse mycotoxins on gut microbiota and the counteracting capabilities of Nano-Se.

## Data availability statement

The datasets presented in this study can be found in online repositories. The names of the repository/repositories and accession number(s) can be found at: https://www.ncbi.nlm.nih.gov/, PRJNA934281.

## Ethics statement

The animal study was approved by the Animal Care and Use Committee of Yichun University, Yichun, China. The study was conducted in accordance with the local legislation and institutional requirements.

## Author contributions

MF and WH were responsible for the study conception and design. MF and BL revised the manuscript. MF, WH, and BL were involved in drafting the manuscript. All authors contributed to the article and approved the submitted version.

## Funding

This work was supported by the Science and Technology Project of the Jiangxi Provincial Department of Education (GJJ211632) and the Initial Scientific Research Fund of Yichun University (3360119046).

## Conflict of interest

The authors declare that the research was conducted in the absence of any commercial or financial relationships that could be construed as a potential conflict of interest.

## Publisher’s note

All claims expressed in this article are solely those of the authors and do not necessarily represent those of their affiliated organizations, or those of the publisher, the editors and the reviewers. Any product that may be evaluated in this article, or claim that may be made by its manufacturer, is not guaranteed or endorsed by the publisher.

## Footnotes

^1^ https://cloud.majorbio.com/

^2^ http://www.hmdb.ca/

^3^ https://metlin.scripps.edu/

^4^ https://www.kegg.jp/kegg/pathway.html
